# Analyzing the genomic variation of microbial cell factories in the era of “New Biotechnology”

**DOI:** 10.5936/csbj.201210012

**Published:** 2012-11-15

**Authors:** Markus Herrgård, Gianni Panagiotou

**Affiliations:** a Novo Nordisk Foundation Center for Biosustainability, Technical University of Denmark, DK-2970 Hørsholm, Denmark; b Center for Biological Sequence Analysis, Department of Systems Biology, Technical University of Denmark, DK-2800 Kongens Lyngby, Denmark; c School of Biological Sciences, The University of Hong Kong, Pokfulam Road, Hong Kong

## Abstract

The application of genome-scale technologies, both experimental and *in silico*, to industrial biotechnology has allowed improving the conversion of biomass-derived feedstocks to chemicals, materials and fuels through microbial fermentation. In particular, due to rapidly decreasing costs and its suitability for identifying the genetic determinants of a phenotypic trait of interest, whole genome sequencing is expected to be one of the major driving forces in industrial biotechnology in the coming years. We present some of the recent studies that have successfully applied high-throughput sequencing technologies for finding the underlying molecular mechanisms for *(a)* improved carbon source utilization, *(b)* increased product formation, and *(c)* stress tolerance. We also discuss the strengths and weaknesses of different strategies for mapping industrially relevant genotype-to-phenotype links including exploiting natural diversity in natural isolates or crosses between isolates, classical mutagenesis and evolutionary engineering.

## Introduction

The field of industrial biotechnology requires rapid and efficient microbial cell factory design and construction for production of fuels, chemicals, proteins and pharmaceuticals. Modern strain design process starts with the identification of a set of genetic target modifications anticipated to improve yield, productivity, robustness or other performance relevant features. Subsequently, the whole arsenal of analytical techniques available today to characterize the transcriptome, metabolome, proteome and other -omes of production strains helps to identify opportunities for additional rounds of metabolic engineering. However, capabilities for strain design and construction have dramatically expanded due to advances in DNA synthesis and sequencing, moving from narrow studies focused on a few genes to broad and deep searches that seek to optimize traits at the genome-scale level (Lewis *et al*., 2012). Despite these advances, our incomplete understanding of the genetic basis of complex cellular processes makes target identification a key challenge in strain engineering.

Metabolic engineering, which integrates engineering design with systematic and quantitative analysis of metabolic pathways, has as central goal to identify gene targets for the optimization of the metabolic phenotype with an emphasis on the global state of the cell, and not the individual reactions (Tyo et al., 2007; Zommorodi et al., 2012). Often rational metabolic engineering is complimented by mutagenesis and screening or by evolutionary engineering, where selective pressure is applied to confer a desirable phenotype (Oud et al., 2012). The problem with microbial strains that have been improved by classical mutagenesis or evolutionary processes is that the exact genetic modification or resulting genotype that leads to the improved phenotype is often not identified or understood. The knowledge of the exact genotype of the strains is vitally important for further rational metabolic engineering as well as securing intellectual property related to production strains.

**Figure 1:**
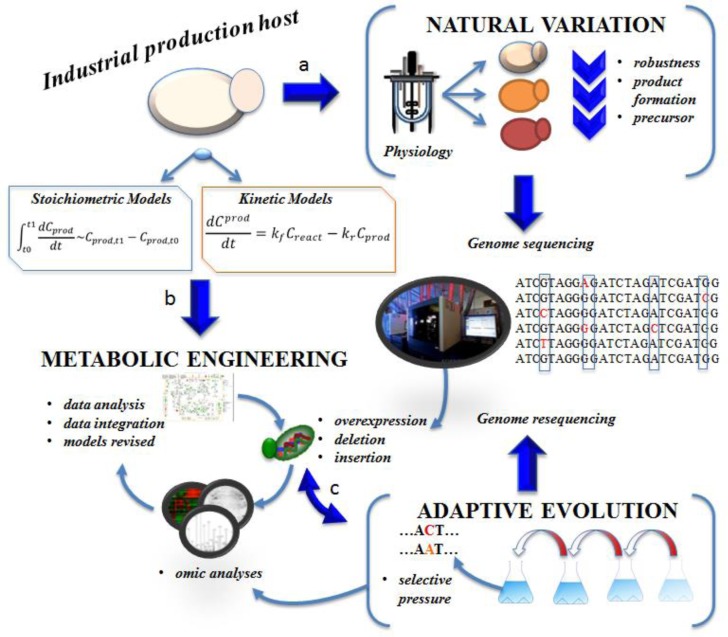
**Optimization of microbial metabolic pathways in industrial biotechnology is focused on physiological parameters, such as maximum specific growth rate, substrate consumption rates, product yields and titers, by-product formation, and morphology.** (A) The optimization process relies on the host′s natural variation. Physiological characterization of natural variants of the production host could reveal phenotypes of interest (increased product formation, tolerance against toxic compounds, elevated concentrations of precursor molecules). Genome wide sequencing of the variants can be used for identifying regulatory points responsible for the observed phenotype and subsequently for designing genetic engineering strategies in the form of overexpression, deletion or insertion. (B) The optimization through metabolic engineering strategies requires the reconstruction of the genome scale metabolic network of the production host. The genome scale metabolic network can be used for developing stoichiometric or kinetic models, which can be applied for predicting gene overexpressions, deletions, or non-native pathway reconstructions. Once a metabolic engineering strategy with high-probability of success has been identified genetic engineering is performed yielding a modified strain. The modified strain is initially characterized often requiring transcriptome, proteome and metabolome measurements, and this analysis should lead to a revised model with improved predictive power. (C) Adaptive evolution is another strategy for optimization of metabolic pathways both as a stand-alone method or coupled with rational approaches. The modified strain from a metabolic engineering strategy can further undergo directed evolution or other non-targeted approaches to yield an improved phenotype. The evolved mutants with desirable phenotypes are characterized by the use of multi-omics while genome re-sequencing reveals the beneficial mutations that can be reintroduced to the production host.

Tools from systems biology (Figure 1) have offered new opportunities for establishing links between genotype and phenotype and, hereby, allow for combinations of random and rational approaches to strain improvement (Boyle and Gill, 2012). However, what has revolutionized the field is the ability to perform deep sequencing of several microbial cell factories with decreasing cost to efficiently search genome-wide spaces for genes conferring desired phenotypes (Kahvejian et al., 2008; Morozova and Marra, 2008). Even though transcriptome, metabolome, proteome or other omics have extensively used, often with great success, for the identification of relevant genetic changes, whole genome sequencing is superior to the other analytical techniques for directly identifying the sequence changes that give rise to specific phenotypes. The independency of genome sequences from the experimental conditions and the possibility to directly and precisely reproduce the identified changes in production hosts is one of the key advantages over gene/protein expression analysis (Warner et al., 2009). In this review we present some of the recent studies that have successfully applied new high-throughput sequencing technologies for finding the underlying molecular mechanisms for a derived or natural phenotype for metabolic engineering applications.

## Reverse metabolic engineering for industrially important metabolic traits

Whereas the conventional ′forward′ metabolic engineering cycle starts with a knowledge-based design, which is then tested by construction of relevant strains, the reverse metabolic engineering cycle starts with existing microbial strains with superior phenotypic traits compared to reference strains. These strains with better phenotypes could either derive from natural sources or may have been created through non-targeted strain improvement efforts. In this section we will highlight the studies that have paved the way in the utilization of whole genome sequencing to screen for genes responsible for desired phenotypes and more specifically understand the genetic basis of, (a) carbon source utilization, (b) product formation, and (c) stress tolerance.

### Improved carbon source utilization

The series of studies published in the past 10 years on laboratory adaptation of *Escherichia coli* to efficiently utilize glycerol, a possible alternative carbon feedstock derived from biodiesel processes (Ibarra et al., 2002), highlight many of the relevant issues related to identifying the genetic basis of industrially interesting phenotypes. The initial study by Ibarra *et al*. (2002) showed that the physiological end-points of adaptive laboratory evolution experiments could be predicted by genome-scale metabolic models through optimization approaches. In order to identify the mechanisms of adaptation evolved strains were initially characterized by gene expression profiling (Fong et al., 2005), which revealed that while the phenotypes of different adaptive lineages were convergent and reproducible, the gene expression states were highly divergent and involved large numbers of changes from the reference wild type strain.

When whole genome re-sequencing became affordable, six clones from the glycerol evolution studies were re-sequenced and a total of 13 mutations were identified and their causative roles were verified by creating site-directed mutants (Herring et al., 2006). Mutations with pleiotropic effects such as those found in the subunits of the RNA polymerase were found to be underlying the broad changes in gene expression observed previously. Specific mutants from the re-sequencing study were further characterized at the biochemical level in follow-up studies (Conrad et al., 2010; Applebee et al., 2011). For example mutations in the *rpoC* subunit of the RNA polymerase were found to alter the kinetics of the transcription process resulting in a 10-fold decrease in transcriptional pausing (Conrad et al., 2010). In parallel, quantitative proteomic profiles of the evolved strains grown on glycerol were measured, and the profiles were found to be consistent with enzyme usage in optimal growth state computations using genome scale metabolic models (Lewis et al., 2010). This series of studies has demonstrated that physiological endpoints of evolutionary adaptation can be predicted by genome-scale metabolic models, but the specific molecular mechanisms of adaptation can be quite unpredictable.

In recent years, other studies on adaptation to suboptimal carbon sources have been performed in both *E. coli* and other bacteria. In an attempt to investigate the genetic basis of adaptive evolution of *E. coli* on a non-native carbon source, Lee *et al*. (Lee and Palsson, 2010), isolated a mutant able to grow on L-1,2-propanediol (L-1,2-PDO). They characterized the evolved *E. coli* mutant that had a growth rate of 0.35 h^-1^ using L-1,2-PDO as a sole carbon and energy source. Using whole-genome sequencing the authors identified all the accumulated mutations providing insights into the genetic basis underlying microbial evolution for growth on a non-native substrate. A SNP found in the *fucO* gene, which is involved in the first step of L-1,2-PDO catabolism in *E. coli*, appears to have allowed the evolved strain to overcome metal-catalyzed inactivation and utilize L-1,2-PDO.

Summers and colleagues (Summers et al., 2012) evaluated the ability of *Geobacter sulfurreducens* to adapt for faster growth on lactate, a common bioremediation amendment. Serial transfer of cultures in a medium with lactate as the sole electron donor yielded strains adapted for rapid metabolism of lactate, and whole-genome sequencing revealed that all evolved strains had non-synonymous SNPs (nsSNPs) in the same gene. With the exception of *GSU0514*, a putative transcriptional regulator, no mutations in other genes were detected, demonstrating that a single base-pair change resulting in a non-synonymous change in amino acid can markedly influence the metabolic phenotype of *G. sulfurreducens*. The authors verified their hypothesis by introducing the single-base-pair mutation into the wild type and monitoring the growth on lactate.

The study of Hong *et al*., (Hong et al., 2011) is probably the best example of integrating adoptive evolution with systems biology for identifying the underlying molecular mechanisms for improved carbon source utilization phenotype in a eukaryotic system. The authors analyzed the changes in transcriptome, metabolome and genome sequence of a yeast strain evolved to rapidly grow on galactose and related these changes to the acquired phenotypic properties. The genomes of the three mutants that had 24% faster growth rates on galactose were sequenced and compared to the reference strain. Even though about one third of the SNPs were in coding regions, no mutations were detected in galactose regulatory and structural genes including *PMG2*, which is considered based on literature data as the most beneficial target for increasing the galactose uptake rate. Only genes encoding proteins of the Ras/PKA signaling pathway were found to carry mutations in all three evolved mutants, which when reconstructed in the wild type resulted to 10% higher specific growth rate. By integrating the genomic information of the evolved strains with the -omic characterization the authors demonstrated that adaptive evolution results in the utilization of unexpected routes to accommodate increased galactose flux.

Using a different approach Otero *et al*., (Otero et al., 2010) performed whole genome sequencing and annotation to identify SNPs between the comprehensively annotated reference genome for the S288C laboratory strain (www.yeastgenome.org) and CEN.PK113-7D, a preferred laboratory strain for industrial biotechnology research. Considering only metabolic genes the authors detected a total of 85 metabolism-specific non-synonymous SNPs distributed across 158 metabolic genes with clear correlations between physiology and metabolic pathway enrichment. Specifically the non-synonymous SNP enrichment in *GAL1* and *GAL10* was correlated with the lower galactose uptake rate of S288c compared to CEN.PK113-7D suggesting obvious targets for improving galactose respiro-fermentative metabolism in S288c. In a parallel study, Nijkamp *et al*., (2012) uncovered genes in CEN.PK113-7D that are absent in S288c and relate to maltose metabolism and biotin biosynthesis of CEN.PK113-7D, which led to the surprising discovery that CEN.PK113-7D is a biotin prototroph, a phenotypic trait potentially interesting for industrial applications.

### Increased product formation

Madsen *et al*., (Madsen et al., 2011) applied an array of computational tools in order to show that the nsSNPs in the *Erg8*, *Erg9* and *HFA1* genes that were found in the genome comparison of CEN.PK113-7D and S288c (Otero *et al*., 2011) are responsible for higher ergosterol concentration in CEN.PK. The specific biochemical mechanisms were linked to alterations in protein structure with direct implications in the stability and substrate affinity of the proteins by sequence-based analysis of the nsSNPs effects, comparative modeling of protein structures, free energy differences and stability analysis of native and mutant proteins, and scanning of binding pockets and functional residues. The authors utilized the detected metabolic SNPs for constructing a production platform of triterpenoids, which resulted to a *S. cerevisiae* strain capable of producing 500% more β-amyrin than the control strain.

Comparative genomics strategies for identifying genetic basis of increased product formation can be extended to less well-studied industrial hosts than yeast or *E. coli* as demonstrated by the study of Andersen *et al*. (Andersen et al., 2011). This study presented the complete genome sequence of the acidogenic *Aspergillus niger* wild type strain (ATCC 1015) and compared it with the genome of an industrial enzyme-producing *A. niger* strain (CBS 513.88). The comparison of the two strains revealed an exceptionally high number of SNPs per kilobase, several of which were accumulated in metabolic pathways essential to protein synthesis and acid production. In connection to protein production the proline, aspartate, asparagine, tryptophan and histidine biosynthesis pathways were enriched in mutations. Mutations relevant for the production of citric acid were found in the TCA cycle, electron transport chain, plasma membrane-bound ATPase and the GABA shunt.

Smith *et al*., (Smith and Liao, 2011) constructed an isobutanol producing strain of *E. coli* using random mutagenesis and selection with a toxic valine analog, norvaline. Since higher alcohol biosynthetic pathways utilize the 2-keto acids, which are precursors of native amino acids, this amino acid anti-metabolite selection strategy could be a powerful tool for the construction of higher alcohol producing strains. However, the authors demonstrated that not all strains that exhibited improved norvaline resistance displayed improved isobutanol production. The genomic sequencing of the best producer mutant identified 208 total mutations, several of which were found in a variety of amino acid biosynthetic pathways. A deleterious mutation was identified in *rpoS*, a master regulator of transcription during the cell′s transition to stationary phase. When *rpoS* wild type allele was restored in the mutated strain, it increased the titer of isobutanol (21.2 g/L) and resulted in an isobutanol yield equal to 76% of the theoretical maximum.

In a recent study Charusanti *et al*., (Charusanti et al., 2012) showed that evolutionary engineering could be used to depelop production hosts for more complex metabolites. The authors hypothesized that microorganisms with extensive secondary metabolism could adaptively evolve to synthesize novel antibacterial molecules if they had to compete against a target pathogen. The authors isolated several *Streptomyces clavuligerus* strains by adaptively evolving multiple colonies to efficiently compete against the methicillin resistant *Staphylococcus aureus* (MRSA) strain N315. The method led to identification of a *S. clavuligerus* strain that produces a known antibiotic, holomycin, which is not produced in detectable quantities by the wild type strain. Genome re-sequencing revealed that the evolved strain had lost pSCL4, a large plasmid constituting 21% of the genome content of *S. clavuligerus*. In addition *S. clavuligerus* acquired several SNPs in genes that have been shown to affect secondary metabolite biosynthesis offering a mechanistic explanation to the activation of holomycin production in the evolved strain.

Whole genome sequence comparison between two *Thermoanaerobacter* species was used in combination with flux analysis and microarray experiments to explain not only why the strain X514 produces higher yields of ethanol from xylose compared to the 39E strain but also to explore other significant differences in the physiology of the two strains with respect to ethanol production (Hemme et al., 2011). Compared to 39E, X514 was found to have additional alcohol dehydrogenases and xylose transporters, modifications to pentose metabolism and a completely new vitamin B_12_ biosynthetic pathway. The authors suggested that the employment of xylose-specific *Xyl* transporters in the X514 strain may explain the observed greater absolute flux and the ability of the strain to grow at lower xylose concentrations than those for growth of 39E, whereas the capacity for *de novo* synthesis of vitamin B_12_ by X514 appeared to be a key factor in maintaining high ethanol yields.

### Improved stress tolerance

Atsumi *et al*., (Atsumi et al., 2010) applied a sequential transfer method to identify genotype-phenotype relationships in isobutanol tolerance. By evolving a rationally engineered isobutanol production *E. coli* strain the authors isolated mutants showing increasing tolerance not only to isobutanol but also n-butanol and 2-methyl-1-butanol. The authors used whole-genome resequencing to identify relevant mutations for the tolerant phenotype and detected one SNP, 25 insertion sequence elements, of which the 22 were contained within coding regions, and a large deletion containing 62 genes. They systematically repaired each mutation showing that most of the mutations did contribute to the overall phenotype and that no single repair abolished the tolerance phenotype. Five mutations, *acrA, gatY, tnaA, yhbJ* and *marCRAB*, and the metabolite glucosamine-6-phosphate, were found to be primarily responsible for the increased isobutanol tolerance. It was also interesting that the isobutanol-tolerant mutants did not perform better in terms of final titer of isobutanol production indicating that strain performance in this case was not adversely affected by low product tolerance.

The analysis of the diploid genome of the efficient industrial fuel-ethanol fermentative and highly ethanol tolerant *S. cerevisiae* CAT-1 strain uncovered important sequence and structural variation compared to the S288c reference strain (Babrzadeh et al., 2012). *IRA1* and *IRA2*, two genes that act as inhibitors of the Ras-cAMP-PKA pathway by increasing the rate at which Ras proteins hydrolyze GTP, were likely associated with traits for prevalence and persistence during ethanol fermentations. However, the phenotypic effects for the majority of the identified sequence polymorphisms were unknown and require the sequencing of additional bioethanol yeast for correlating genome sequence data to phenotypic differences.

Starting from a single colony of a wild-type *Clostridium thermocellum* strain and using cellobiose or cellulose as the substrate for growth Shao *et al*., (Shao et al., 2011) evolved this strain for growth with up to 50g/L ethanol. In an effort to unravel the genetic changes associated with ethanol tolerance the authors sequenced the genomes of the mutant strains revealing 10 and 39 non-synonymous SNPs in the E50A (cellulose) and E50C (cellobiose) strains, respectively. Four genes had identical changes in both strains including genes involved in arginine biosynthetic pathways and a putative glucokinase gene. These mutations might be related to minimizing the ethanol inhibitory effect by regulating the steady-state levels of the carbamyl intermediates under the threshold for spontaneous reaction with ethanol. On the other hand the bi-functional aldehyde/alcohol dehydrogenase involved in ethanol production from acetyl-CoA (*adhE*) and aspartate carbamoyl transferase involved in pyrimidine biosynthesis from carbamoyl phosphate (*pyrB*) were independently mutated in the two strains. Using homology-based structural modeling the authors suggested that the location of the altered amino acids residues with the protein structures of *AdhE* suggest possible alterations in co-factor specificity, catalytic efficiency and/or stability although this needs to be tested with further experiments.

Most of the studies discussed previously only used sequence data for a handful of either wild isolates or isolates obtained from adaptation or selection studies. With the availability of low cost sequencing technologies these types of studies can now be scaled up in terms of the number of candidate mutations identified. This type of scale-up in sequencing volume is exemplified by the Tenaillon *et al*. (Tenaillon et al., 2012) study that sequenced the genomes of 115 distinct *E. coli* isolates obtained from an equal number of independent experimental evolutions at an elevated growth temperature. The authors identified a total of 1331 mutations, and thus characterized a large number of alternative genetic strategies for adaptation to higher growth temperature. A common core set of mutation targets such as the RNA polymerase was identified although the specific mutations tended to be different in each of the isolates. The large number of isolates sequenced also allowed using statistical methods for identifying putative epistatic interactions between mutation targets suggesting which combinations of mutations most parsimoniously reconstitute the temperature tolerance phenotype.

As an alternative to direct sequencing of large numbers of isolates described above, Ehrenreich *et al*., (Ehrenreich et al., 2010) applied a novel approach called extreme QTL mapping (X-QTL) to a cross between a laboratory strain and a wine strain of *S. cerevisiae* in order to identify the genetic determinants of chemical tolerance in very large mapping populations. The X-QTL mapping approach is based on (1) creating a very large pool of segregants from a cross, (2) selecting a specific segregant population from this pool that is resistant to e.g. chemical stress, and (3) bulk estimation of allele frequencies in this pool by either microarrays or next generation sequencing. When the X-QTL approach was used with 16 different chemical resistance traits including ethanol and oxidative stress (paraquat) tolerance, it identified between 1 and 24 major genetic loci controlling each trait. The study was later extended to 6 pairwise crosses of additional *S. cerevisiae* strains to identify whether observed the genetic complexity of traits is mainly dependent on the trait or on the genetic background of the parent strains (Ehrenreich et al., 2010). While the overall genetic complexity depended on the trait, each additional cross also contributed a significant number of additional genetic loci over the existing crosses. Taken together the results from the X-QTL studies indicate that chemical resistance traits in yeast are controlled by a relatively large number of genetic loci (8 to 57) and that both rare and common allelic variants contribute to resistance. Additional work will be required to verify at the individual SNP level the determinants of resistance identified by X-QTL, but the approach itself provides a rapid way to comprehensively identify interesting genetic variants for practical applications.

## Discussion

Genotype-to-phenotype relationships relevant for industrial strain development can be mapped based on genomic sequence information on strains that either have been isolated from natural sources, or have been derived by classical mutagenesis, evolutionary engineering or crosses between strains. Each source of genetic variation has its own upsides and drawbacks with respect to identifying genotype-phenotype links, which we discuss below.

The large number of wild-type strains present in the environment and in public and private culture collections are an important source of natural diversity of industrial phenotypes (van Hylckama Vlieg et al., 2006). This biodiversity can now be exploited for industrial innovations using genomics and high-throughput technologies. Furthermore, genetic markers for complex phenotypes can be identified by correlating the gene content to a variable trait (Pretzer et al., 2005). These approaches generate genetic markers that can then be targeted in miniaturized screening approaches to allow the rapid identification of a strain exhibiting the desired properties (Smit et al., 2004). However, the major disadvantage of such approaches is that often natural diversity might not provide us with strains with desired properties, simply because such phenotypes are not competitive in natural niches. This lack of a dominating and unique phenotype adds another level of complexity to the large number of SNPs that is usually found between different wild type strains, making hard to explain their contribution to a single phenotype.

Classical strain development relies on generation of genetic diversity through mutagenesis using classes of mutagens with different modes of action such as radiation, UV rays, chemicals, intercalating agents, and other biological agents. The result of a typical mutagenesis program is random damage to the DNA through strand breakage, transversion, addition, deletion, or substitution of bases and it is followed by selection or screening of colonies exhibiting the desired phenotypes. Although classical approaches for creating superior strains have their niche they suffer for being a slow laborious process especially for phenotypes that are dependent on multiple coordinated changes at the genetic level (Patnaik, 2008). Furthermore, the screening of large combinatorial libraries for successful isolation of desired polygenic phenotypes is practically impossible to implement. Whereas phenotypic screenings for improved growth or stress tolerance can be relatively easily designed, screening for mutants with increased product formation is only possible in special cases where anti-metabolites can be used. Most mutagenesis strategies also create significantly more genetic changes that are actually needed to realize the desired phenotype making it difficult to map genotype-to-phenotype relationships by sequencing. As demonstrated by the novaline selection study, random mutagenesis can also generate deleterious mutations that significantly reduce strain fitness in standard cultivating conditions.

Evolutionary engineering uses a selection pressure and allows the microorganism to evolve naturally in a chemostat, through sequential batch propagation, or in plate propagation, and thus it relies on the inherent capacity of the cell to introduce adaptive mutations (Sauer, 2001). Fast generation times, repeatability, the ease of maintaining large population sizes, and the ability to store populations for later examination are microbes′ characteristics that make them well suited for laboratory evolution (Elena and Lenski, 2003). However, without automation this method is also laborious and time consuming due to extensive cultivations periods that are required for the successful selection of the desired phenotypes with the limited natural mutation rates that most microbial species exhibit. Furthermore, the identification of these mutations that are necessary for conferring a particular phenotype is still not straightforward, since neutral mutations tend to accumulate therefore a full ′′omic′′ analysis may be required to understand the basis for the observed phenotypic differences (Bro and Nielsen, 2004). However, for several reasons *in vivo* microbial evolution experiments provide additional power compared with reverse genetics approaches that are based on direct-targeted activation or inactivation of genes. While in the reverse genetics the phenotypic screens are based on a ′′ plus (positive) or minus (negative) ′′ effect as a result of gene inactivation or modification of residues that cause severe alteration in protein functionality, evolution experiments can select for mutations with more subtle effects providing opportunities for finding novel functions or functional domains in genes and proteins. Additionally, the temporal order in which mutations arise can be easily established in evolutionary engineering studies by sequencing isolates or populations at different time points. This information can be correlated with temporal order of phenotypic changes allowing establishing a putative causative role of specific mutations as well as epistatic relationships between mutations.

Classical genetics in the form of crosses and QTL analysis has not been used extensively in microbial cell factory engineering applications both due to lack of familiarity with genetics methods among strain engineers and also due to limited resolution of traditional QTL mapping techniques. However, modern mapping methods like X-QTL are significantly faster than classical methods, and coupled with next generation sequencing have also potential for base pair-level resolution. One of the major benefits in crossing approaches is that the starting genetic variation is controlled as the parental strains can be genotyped, and mature statistical genetics tools can be used to identify and prioritize major variants. Crosses are also the only comprehensive and unbiased way to map the phenotypic effects of genetic changes between two strains that differ at significant numbers of loci. The major drawback is that the approach is limited to sexually reproducing microbes and in particular to microbes where haploid progeny from a cross can be easily isolated like yeast. Crosses can of course also only sample limited genetic variability, as the mutations have to be present in one of the parental strains.

**Figure 2:**
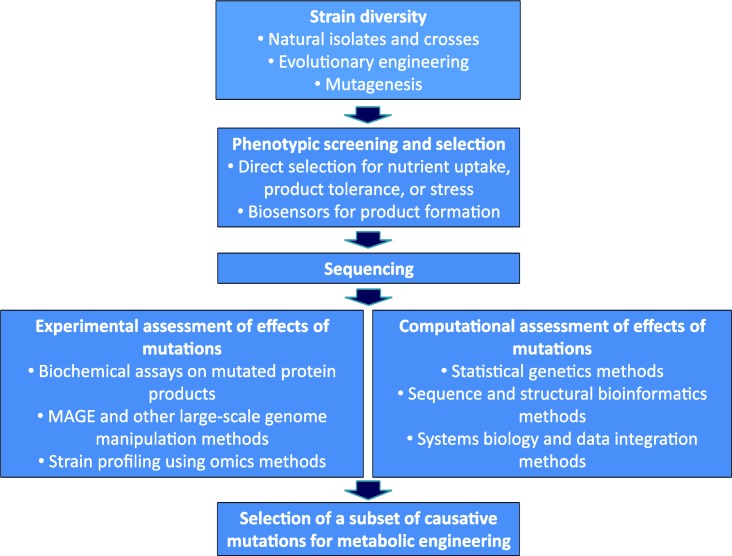
**Modern workflow for the identification of new genetic variants for metabolic engineering applications.** The sequencing component of this workflow is no longer the bottleneck in the process, but all other components have to be made more efficient and the entire process has to be integrated as a routine part of all metabolic engineering projects.

Despite impressive progress in methods for mapping the genetic basis of industrially relevant phenotypes, there is still need to speed up the process of finding and verifying genotype-metabolic phenotype links (Figure 2). The process of evolutionary engineering needs to be further automated to allow generating larger numbers of strains with variable genotypes but equally optimal phenotypes (Conrad et al., 2011). Methods for selection of relevant phenotypes by using for example biosensors that target specific metabolic precursors of interest for a broad category of products like malonyl-CoA or mevalonate are also urgently needed (Zhang and Keasling, 2011). Further decrease in sequencing cost will also allow increasing the number of individual isolates from a single selection experiment that can be genotyped. Increased sequencing capacity can of course also be applied to genotyping existing large collections of wild isolates for relevant production organisms such as yeasts.

All these developments allow to determining an increasing number of genetic variants underlying metabolic phenotypes, but do not help in verifying the causative effects of these variants or selecting a subset of them for practical use in production strains. Ideally all combinations of high confidence mutations linked to phenotypes of interest discovered in the genotyping studies would be tested in clean wild type backgrounds using high throughput genetic modification techniques such as multiplex automated genome engineering (MAGE) or other recombineering approaches (Wang et al., 2009). These methods are very promising, but currently they are still limited to testing only relatively small numbers of simultaneous combinatorial mutations (Sandoval et al., 2012) and it is unlikely that for example all combinations of all the 1600+ mutations discovered in the *E. coli* temperature adaptation study could be constructed in a reasonable time frame.

With these challenges in mind, bioinformatics methods similar to those that are being employed increasingly in modern next generation sequencing-based human genetics can also contribute significantly in prioritizing genetic changes that need to be experimentally tested. These methods include enhanced approaches for predicting effects of mutations protein structure or expression based on sequence or structural features (Francesconi et al., 2011). Mutations can also be prioritized by improved genetic mapping methods that utilize not only large collections of individuals for which both genotypes and phenotypes have been determined, but also network information in the form of metabolic, regulatory and protein interaction networks. For well-studied organisms like yeast and *E. coli* more predictive models of e.g. metabolic networks can be used as a scaffold for interpreting and prioritizing genotyping data (Lewis et al., 2012). The ultimate goal of systems biology is to be able to model the effects of mutations in relevant genetic components of the system of interest on phenotypic characteristics, and these same approaches can of course also be used to identify which mutations of the ones that have been discovered by sequencing would be expected to have biggest contribution to phenotypes of interest. In order to achieve this goal we need methods that combine mechanistic modeling of known biochemical processes (Karr et al., 2012; Lerman et al., 2012; Thiele et al., 2012; Lee et al., 2012) and statistical modeling of genotype-phenotype relationships (Jelier et al., 2011).

Impressively rapid developments in sequencing technologies have revolutionized fields like human genetics and epigenetic studies. These same developments can also help to revolutionize microbial cell factory engineering by providing a more solid and comprehensive genetic basis for engineering efforts. However, sequencing alone will only provide the starting point for cellular engineering and significant advances in synthetic biology techniques, bioinformatics and modeling, as well as high-throughput phenotypic screening will be needed to realize the potential of increased sequence information.
